# An Evaluation of Acylated Ghrelin and Obestatin Levels in Childhood Obesity and Their Association with Insulin Resistance, Metabolic Syndrome, and Oxidative Stress

**DOI:** 10.3390/jcm5070061

**Published:** 2016-06-23

**Authors:** Maryam Razzaghy-Azar, Mitra Nourbakhsh, Abdolreza Pourmoteabed, Mona Nourbakhsh, Davod Ilbeigi, Mohsen Khosravi

**Affiliations:** 1 Metabolic Disorders Research Center, Endocrinology and Metabolism Molecular–Cellular Sciences Institute, Tehran University of Medical Sciences, 1411715851 Tehran, Iran; mrazar@tums.ac.ir; 2 H. Aliasghar Hospital, Iran University of Medical Sciences, 1449614535 Tehran, Iran; a_p_808@yahoo.com (A.P.); dr.mnrbh@yahoo.com (M.N.); 3 Department of Biochemistry, School of Medicine, Iran University of Medical Sciences, 1449614535 Tehran, Iran; dara1khosravi@gmail.com; 4 Department of Biochemistry, School of Medicine, Tehran University of Medical Sciences, 1417614418 Tehran, Iran; ildavod@yahoo.com

**Keywords:** ghrelin, obestatin, obesity, insulin resistance, metabolic syndrome, oxidative stress

## Abstract

Background: Ghrelin is a 28-amino acid peptide with an orexigenic property, which is predominantly produced by the stomach. Acylated ghrelin is the active form of this hormone. Obestatin is a 23-amino acid peptide which is produced by post-translational modification of a protein precursor that also produces ghrelin. Obestatin has the opposite effect of ghrelin on food intake. The aim of this study was to evaluate acylated ghrelin and obestatin levels and their ratio in obese and normal-weight children and adolescents, and their association with metabolic syndrome (MetS) parameters. Methods: Serum acyl-ghrelin, obestatin, leptin, insulin, fasting plasma glucose (FPG), lipid profile, and malondialdehyde (MDA) were evaluated in 73 children and adolescents (42 obese and 31 control). Insulin resistance was calculated by a homeostasis model assessment of insulin resistance (HOMA-IR). MetS was determined according to IDF criteria. Results: Acyl-ghrelin levels were significantly lower in obese subjects compared to the control group and lower in obese children with MetS compared to obese subjects without MetS. Obestatin was significantly higher in obese subjects compared to that of the control, but it did not differ significantly among those with or without MetS. Acyl-ghrelin to obestatin ratio was significantly lower in obese subjects compared to that in normal subjects. Acyl-ghrelin showed significant negative and obestatin showed significant positive correlations with body mass index (BMI), BMI *Z*-score, leptin, insulin, and HOMA-IR. Acyl-ghrelin had a significant negative correlation with MDA as an index of oxidative stress. Conclusion: Ghrelin is decreased and obestatin is elevated in obesity. Both of these hormones are associated with insulin resistance, and ghrelin is associated with oxidative stress. The balance between ghrelin and obestatin seems to be disturbed in obesity.

## 1. Introduction

The prevalence of obesity in children is increasing worldwide. The American Obesity Association estimates that 30% of children between the ages of 6 and 19 are overweight, and 15% meet the criteria for obesity [1]. There is ample evidence suggesting that the complications of obesity seen in adults, begin in early childhood [2]. Insulin resistance (IR), hypertension, glucose intolerance, and type 2 diabetes mellitus have all been associated with childhood obesity [3]. The concomitant occurrence of these abnormalities, referred to as metabolic syndrome (MetS), accrues a significant risk for the development of cardiovascular disease and diabetes during early adulthood, if not during childhood. Non-alcoholic fatty liver disease is also another consequence of obesity that is accompanied with a higher rate of mortality compared to general population [4].

Investigations have focused on two gastric hormones, ghrelin and obestatin, and their role in the regulation of food intake in the children with obesity. Ghrelin and obestatin exert a marked effect on the motivation to eat and food intake, and are modulated during puberty by habitual physical activity levels. Ghrelin is a 28-amino acid peptide predominantly produced by the stomach and expressed in many other central and peripheral tissues [5]. In addition to potent GH-releasing activity, ghrelin is involved in the control of food intake and energy metabolism at central as well as peripheral levels influencing the endocrine pancreatic function and glucose and lipid metabolism [6,7]. The ghrelin-ghrelin *O*-acyltransferase system has recently been found to play a crucial role in both the development of steatosis and its progression to nonalcoholic steatohepatitis [8]. Ghrelin has also been shown to improve antioxidant defense [9]. Obesity is strongly associated with oxidative stress which occurs as a result of imbalance between oxidants and antioxidants. Adipose tissue is the major source of reactive oxygen species (ROS) production, and fat accumulation is closely related to increased oxidative stress. On the other hand, oxidative stress is involved in the induction of IR, and it has been demonstrated to be an early instigator of the obesity-associated development of MetS [10].

Ghrelin circulates in two forms, simply differing in the acylation, the most abundant of which is non-acylated ghrelin, while the acylated form is considered to be the active hormone [7]. It has been demonstrated that acylation is essential for the binding of ghrelin to the GHS receptor in the hypothalamus and the pituitary, and consequently for its functions [11].

Several in vitro and in vivo studies have shown that ghrelin may regulate insulin and glucose metabolism [12]. Studies in humans have shown that circulating levels of ghrelin are increased in anorexia and cachexia, but are reduced in obesity, insulin resistance (IR), and type 2 diabetes [6,11,13,14].

Obestatin, a 23-amino acid peptide, is produced by the post-translational modification of a protein precursor that also produces ghrelin and is mainly found in the stomach. Studies in animal models have shown that the effect of obestatin on food intake is opposite to ghrelin, and obestatin has been suggested to provide a new target for the control of obesity [15,16]. Both ghrelin and obestatin have been suggested to contribute to obesity-associated IR in MetS [17].

Based on the previous studies and the influence of these hormones on appetite and insulin secretion, we hypothesized that children with obesity and MetS, compared with age-matched controls, would have impairment in acyl-ghrelin and obestatin levels, as well as in acyl-ghrelin-to-obestatin ratios. According to the antioxidant property of ghrelin, its association with oxidative stress was also investigated. Lipid peroxidation, which follows oxidative stress, generates a variety of relatively stable end products, including malondialdehyde (MDA) which is increased in various disease states [18] and has been considered as a reliable marker of oxidative stress in clinical situations [19]. Therefore, the relationship between ghrelin and MDA was also evaluated.

## 2. Experimental Section

### 2.1. Subjects

Seventy-three children and adolescents, aged 7–16 years, were included in this study. Obesity was defined as a body mass index (BMI) above the 95th percentile for age and sex. With this definition, 42 subjects were considered obese. Thirty-one age- and sex-matched lean subjects with BMIs between the 5th and 84th percentiles were also included as the control group. The sample size was calculated on the basis of the results of a previous study [20], with 80% power and a confidence interval of 95% using PASS software (version 11). 

A thorough history and physical examination was obtained for all the subjects. Apart from obesity, no other clinical or laboratory signs of systemic illness or endocrine perturbation, including diabetes mellitus and hypothyroidism, were observed in obese subjects. Control subjects were selected from those referred to outpatient clinics for check-up and growth evaluations and found to be completely healthy. None of the subjects were receiving medications or supplements other than conventional multivitamins. They had normal physical activity and were not on any special diets. Taking into account the above considerations, case and control subjects were carefully selected and matched.

Anthropometric measurements were performed in the morning after an overnight fast. Weight was determined using a digital electronic weighing scale, with subjects being bare-footed and wearing light clothes. Height was determined in the upright position using a digital stadiometer. Body mass index (BMI) was calculated as kg/m^2^, and BMI *z*-score was determined for all subjects. Blood pressure (BP) was measured by mercury sphygmomanometer. Waist circumference (WC) was measured at the end of a normal expiration, with the measuring tape positioned at the level of noticeable waist narrowing or at the level of the lower floating rib, while the subjects were in a standing position. WC percentiles were determined according to age and gender of each subject [21]. Pubertal staging was determined by Tanner’s classification [22].

Written informed consent was obtained from subjects or their parents or both, and the study was approved by the ethics committee of Tehran University of Medical Sciences.

### 2.2. Metabolic Syndrome and Insulin Resistance Determination

Homeostatic model assessment of insulin resistance (HOMA-IR) was used as an index of insulin resistance (IR). It was calculated by the formula: serum insulin (µIU/mL) × FPG (mg/dL)/405 [23]. Those with HOMA-IR-values greater than 3.16 were classified as having IR [24].

The IDF consensus definition of MetS in children and adolescents was used for the diagnosis of MetS [25]. According to this criteria, children and adolescents with abdominal obesity (WC above 90th percentile for age and sex), and presenting two or more other clinical features of MetS including elevated glucose, TG, BP, or low HDL-C, were classified as having MetS.

### 2.3. Biochemical Measurement

Venous blood samples were collected in the morning after an overnight fast (12 h). Serum and plasma samples were separated and stored at −80 °C until analysis. Fasting plasma glucose (FPG), triglyceride (TG), total cholesterol (TC), high-density lipoprotein cholesterol (HDL-C), and low-density lipoprotein cholesterol (LDL-C) were measured by colorimetric methods using commercial kits (Pars Azmoon, Tehran, Iran).

Serum MDA levels were measured by a colorimetric method using trichloroacetic acid and thiobarbituric acid [26]. The absorbance of the resulting red compound was determined at 535 nm, and MDA concentrations were expressed as mmol/L.

Plasma concentrations of insulin were determined by an enzyme-linked immunosorbent assay (ELISA) using a commercial assay kit (Monobind Inc., CA, USA). Plasma leptin levels were also determined by an ELISA kit (Orgenium, Vantaa, Finland). Acyl-ghrelin assessment was carried out in plasma using an enzyme immunometric assay (EIA) (DRG, Marburg, Germany) by a double-antibody sandwich technique. Plasma obestatin was also measured by an EIA kit (Sigma-Aldrich, Munich, Germany).

### 2.4. Statistical Analysis

Normal distribution of data was evaluated by a Kolmogorov–Smirnov test. Data are presented either as the mean ± standard deviation (SD) or the median (interquartile range). The differences between the patients and the control group were analyzed using the Mann–Whitney *U* test (non-parametric) and independent-samples *t*-test (parametric). The statistical differences were considered significant if the *p*-value was lower than 0.05. Correlations between variables were evaluated using Pearson’s and Spearman’s correlation tests for parametric and non-parametric variables, respectively. To control for confounding factors, partial correlation was used. All the statistical analyses were performed using SPSS software version 16.0 (SPSS, Inc., Chicago, IL, USA).

## 3. Results

The anthropometric and biochemical characteristics of the studied subjects are shown in Table 1. Gender distribution and age of the subjects were not significantly different between the two studied groups. The obese group had significantly higher BMIs, BMI *z*-scores, and WCs compared to normal-weight children and adolescents. Sixty-two percent of obese subjects had WCs above the 95th percentile. Systolic BP was significantly higher in obese subjects than that in normal subjects.

FPG, TG, TC and HDL-C were not significantly different among the studied subjects; nevertheless, obese children and adolescents had significantly higher levels of LDL-C. Compared to control subjects, leptin levels in obese subjects were also significantly higher.

Plasma Ac-ghrelin levels were significantly lower than those of normal-weight children. On the other hand, obestatin concentrations were significantly higher than those of normal-weight children. There were no gender-specific differences in the assessed variables. Furthermore, no differences between the pre-pubertal and pubertal stages were observed.

Compared to control subjects, obese subjects had significantly higher insulin levels and HOMA-IR. Based on HOMA-IR, 61% (*n* = 22) of obese subjects had insulin resistance (IR). None of the control subjects were insulin-resistant. Both Ac-ghrelin and obestatin levels were significantly different in subjects with or without insulin resistance. Ac-ghrelin levels were lower in subjects with IR compared to those without IR (59 (18–75) pg/mL vs. 74 (53–149) pg/mL, respectively) (*p* < 0.01), and obestatin levels were significantly higher in subjects with IR compared to those without IR (246 (155–437) pg/mL vs. 199 (134–275) pg/mL, respectively) (*p* < 0.05).

Normal subjects did not have the features of MetS. On the contrary, MetS was diagnosed in 33% of obese children and adolescents. Both Ac-ghrelin and obestatin levels were analyzed in obese subjects with or without MetS; among them, Ac-ghrelin levels were found to be significantly lower in obese children with MetS compared to obese children without MetS (22.65 (14.9–64) pg/mL vs. 60.0 (22.04–70.0) pg/mL, respectively) (*p* < 0.01). Obestatin levels were not significantly different with regard to MetS.

The correlations between Ac-ghrelin, obestatin, and other variables are summarized in Table 2. Significant negative correlations were found between Ac-ghrelin and BMI, BMI *z*-score, TG, LDL-C, HDL-C, insulin, HOMA-IR, and leptin. On the other hand, Ac-ghrelin showed significant negative correlations with BMI, BMI *z*-score, LDL-C, insulin, HOMA-IR, and leptin, as well as MDA. Nevertheless, a partial correlation analysis with BMI or BMI *z*-score as the confounding factor did not give any significant correlation between studied parameters.

MDA, which is considered an index of lipid peroxidation and oxidative stress, was significantly higher in obese children and adolescents compared to normal-weight subjects.

Ac-ghrelin-to-obestatin ratios were also calculated. This ratio was significantly lower in obese subjects compared with control subjects (Table 1), but it was not different among obese subjects with or without MetS.

## 4. Discussion

Obesity is the result of complex interactions among genes, dietary intake, physical activity, and the environment. Obesity is a critical risk factor for the development of insulin resistance in children [27,28]. Childhood and adolescent obesity leads to development of obesity in adulthood and, eventually, MetS and type 2 diabetes [29].

Ghrelin and obestatin are two secreted peptides which play an important role in the regulation of food intake and body weight [30,31,32]. Several studies have examined the role of ghrelin in obesity. As the administration of ghrelin has shown to increase food intake and fat deposition in some previous reports, ghrelin has been considered as one of the causes of obesity [32,33]. Earlier studies on ghrelin and energy balance and metabolism were performed by total ghrelin assays. However, ghrelin circulates in two forms, simply differing in the acylation, the most abundant of which is nonacylated ghrelin; nonetheless, the most active form is the acylated one [34,35]. In this study, we measured the fasting plasma acyl-ghrelin levels in obese children as well as normal-weight children. In agreement with some previous findings in adults [36] and children [37], we found that the fasting plasma acyl-ghrelin levels in obese children were lower than that in the controls. We also observed significant negative correlations between plasma acyl-ghrelin and BMI, BMI *z*-score, and serum leptin levels. The mechanism by which ghrelin level is reduced in obesity has not been elucidated [6], but our results are in agreement with previous studies that found reduced levels of ghrelin in obesity [38] and type 2 diabetes mellitus [39]. Circulating plasma ghrelin has been previously found to decrease significantly after oral and intravenous glucose administration [14], food intake [40,41], and body weight increase [42]. Therefore, it seems that increased caloric intake suppresses the plasma ghrelin levels and decreased ghrelin may reflect a possible physiological adaptation to positive energy balance.

In this study, we demonstrated that acyl-ghrelin correlated negatively with TG, LDL-C, insulin, and HOMA-IR as components of metabolic syndrome and insulin resistance. Ghrelin and insulin are two hormones which play a relevant role in body weight regulation [43]. Several in vitro and in vivo studies have shown that ghrelin may regulate insulin and glucose metabolism [12,44,45]. Ghrelin was shown to inhibit insulin secretion from pancreatic islets in rodents [45]. Ghrelin secretion may be affected by adiposity through insulin and/or glucose metabolism [46,47]. Studies performed in humans demonstrated that i.v. administration of insulin induces a fall in ghrelin levels [48], although some authors disagree with this [49,50]. Thus, reduced ghrelin levels in obesity may be the consequence of increased insulin levels in these subjects.

Moreover, ghrelin has been shown to be protective against oxidative stress [9]. It reduces lipid peroxidation [51] and normalizes the tissue redox state in high-fat-fed state [52]. On the other hand, studies have shown that obesity in children and adolescents is accompanied by reduced antioxidant potential and high levels of reactive oxygen species which are associated with hypertension and atherosclerosis [10]. The relationship between ghrelin and oxidative stress has not been previously studied in obese children and adolescents. MDA, which is produced as a result of lipid peroxidation, is considered a reliable marker for the assessment of oxidative stress [19]. Here, we showed that a negative significant correlation exists between acyl-ghrelin and MDA, indicating that the protective role of ghrelin in oxidative stress may be diminished in obesity due to the decreased levels of ghrelin.

We measured fasting plasma obestatin levels in obese children as well as normal-weight children. We found that the fasting plasma obestatin levels in obese children were higher than that in the controls, which is in agreement with some previous findings in adults [36]. We also observed a significant positive correlation between obestatin and BMI.

Several studies in animal models have shown that obestatin has the opposite effect of ghrelin, suggesting that it may provide new targets for the control of obesity [15,16,53,54]. It has been reported that ghrelin and obestatin have different glucose-induced dynamic patterns in obese pediatric subjects [17]. The actual role of obestatin in the mechanism of obesity is still not fully understood. Since obestatin has opposing effects to acyl-ghrelin on appetite and weight gain, increased obestatin levels may be an adaptive response to obesity in an attempt to decrease food intake. However, at the same time, obestatin was significantly correlated with both insulin levels and HOMA-IR, but not FPG, indicating an interrelationship between obestatin and insulin. In fact, it has been shown that obestatin potentiated glucose-induced insulin secretion at normal glucose levels, but not at high glucose levels [55], further confirming the observed relationship and the contribution of obestatin with insulin resistance.

Since ghrelin and obestatin are products of a single gene, their levels may be under specific regulation, and the balance between ghrelin and obestatin might play an important role in obesity [53,56,57,58]. Thus, the acyl-ghrelin-to-obestatin ratio was also investigated in obese children and found to be lower in obese children compared to control subjects, indicating a perturbation in the balance between these two peptides. The ratio of total—not acylated—ghrelin to obestatin has been previously investigated in obesity and shown to be lower in obese subjects [59,60].

We demonstrated that, compared to obese children without MetS, plasma acyl-ghrelin levels were significantly lower in obese children with MetS. However, we did not observe a significant difference in obestatin levels in obese children with MetS compared to obese children without MetS. Ghrelin has been shown to be associated with metabolic syndrome components [61], and lean metabolically abnormal children have also shown to have higher acyl-ghrelin levels compared with healthy subjects [62], confirming our findings and indicating a direct association between ghrelin and metabolic syndrome.

## 5. Conclusions

Both ghrelin and obestatin are deregulated in obesity and associated with insulin and insulin resistance and thus may be considered a suitable target for the management of insulin resistance. Ghrelin, but not obestatin, is associated with metabolic syndrome and oxidative stress. The balance between ghrelin and obestatin seems to be an important factor in obesity and its related complications.

## Figures and Tables

**Table 1:** Anthropometric and biochemical characteristics of obese and non-obese children and adolescents.

	Control	Obese	*p* Value
Male/female	10/21	16/26	n.s.
Age (years)	9.7	10.85	n.s.
BMI (kg/m^2^)	17.1 ± 2.4	28.95 ± 6.1	<0.01
BMI *z*-score	−0.1 ± 0.89	2.06 ± 0.5	<0.001
WC (cm)	67.0 ± 8.5	89.4 ± 10.7	<0.01
SBP (mmHg)	104.7 ± 14	122.2 ± 18	<0.05
SBP *z*-score	−0.13 ± 0.6	1.5 ± 0.7	<0.05
DBP (mmHg)	63.7 ± 0.6	73.75 ± 14	n.s.
DPB *z*-score	−0.2 ± 0.3	0.93 ± 1.2	n.s.
FPG (mg/dL)	86.83 ± 10.5	87.95 ± 10.2	n.s.
TG (mg/dL)	74.37 ± 40.4	115.17 ± 59.2	n.s.
TC (mg/dL)	150.28 ± 23.1	170.48 ± 25.3	n.s.
LDL-C (mg/dL)	70.96 ± 10.2	98.51 ± 25.9	<0.01
HDL-C (mg/dL)	45.89 ± 10.2	44.71 ± 10.4	n.s.
MDA (µmol/L)	0.65 ± 0.2	0.81 ± 0.2	<0.05
Insulin (µIU/dL)	9.30 ± 6.5	23.01 ± 15.9	<0.01
HOMA-IR	2.06 ± 1.5	5.2 ± 4.2	<0.05
Leptin (ng/mL)	11.4 ± 10.4	34.55 ± 24.2	<0.001
Ac-Ghrelin (pg/mL)	124.1 (56.28–193.11)	58.1 (18.95–70.0)	<0.001
Obestatin (pg/mL)	180.8 (123.2–214.8)	267 (193.6–450.3)	<0.001

Data are presented as the mean ± SD or median (interquartile range). n.s.: nonsignificant difference; BMI: Body mass index; WC: Waist circumference; SBP: Systolic blood pressure; DBP: diastolic blood pressure; FPG: Fasting plasma glucose; TG: triglyceride; TC: total cholesterol; HDL-C: high-density lipoprotein cholesterol; and LDL-C: low-density lipoprotein cholesterol; Ac-Ghrelin: acylated ghrelin.

**Table 2:** Correlation coefficients of acyl-ghrelin and obestatin with other variables.

	Ac-Ghrelin	Obestatin
BMI (Kg/m^2^)	−0.503 **	0.549 **
BMI *z*-score	−0.266 *	0.311 **
FPG (mg/dL)	0.075	0.106
TG (mg/dL)	−0.258 *	0.190
TC (mg/dL)	−0.011	0.168
LDL-C (mg/dL)	−0.239 *	0.255 *
HDL-C (mg/dL)	0.246 *	0.192
MDA (µmol/L)	−0.398 **	0.201
Insulin (µIU/dL)	−0.434 **	0.308 **
HOMA-IR	−0.402 **	0.282 **
Leptin	−0.322 *	0.642 **

BMI: Body mass index; FPG: Fasting plasma glucose; TG: triglyceride; TC: total cholesterol; HDL-C: high-density lipoprotein cholesterol; and LDL-C: low-density lipoprotein cholesterol; * *p* < 0.05; ** *p* < 0.01.

## Abbreviations

The following abbreviations are used in this manuscript:

MDA	malondialdehyde
FPG	fasting plasma glucose
MetS	metabolic syndrome
HOMA-IR	homeostasis model assessment of insulin resistance
BMI	body mass index
IR	insulin resistance
BP	blood pressure
WC	waist circumference
TC	total cholesterol
TG	triglyceride
HDL-C	high-density lipoprotein cholesterol
LDL-C	low-density lipoprotein cholesterol
